# Noninvasive evaluation of intracranial pressure in patients with traumatic brain injury by transcranial Doppler ultrasound

**DOI:** 10.1002/brb3.2396

**Published:** 2021-11-01

**Authors:** Tao Chang, Xigang Yan, Chao Zhao, Yufu Zhang, Bao Wang, Li Gao

**Affiliations:** ^1^ Department of Emergency The Second Affiliated Hospital of Air Force Medical University Xi'an China; ^2^ Department of Anesthesiology The Second Affiliated Hospital of Air Force Medical University Xi'an China; ^3^ Department of Neurology The Second Affiliated Hospital of Air Force Medical University Xi'an China; ^4^ Department of Neurosurgery The Second Affiliated Hospital of Air Force Medical University Xi'an China

**Keywords:** intracranial pressure, optic nerve sheath diameter, pulsatility index, transcranial Doppler ultrasound, traumatic brain injury

## Abstract

**Introduction:**

The purpose of this study was to investigate the relationship between pulsatility index (PI) or optic nerve sheath diameter (ONSD) and intracranial pressure (ICP) in patients with traumatic brain injury (TBI), and the ability of ONSD and ICP to predict intracranial hypertension.

**Methods:**

A total of 68 patients with TBI were included in this retrospective study. After receiving surgery treatment, they underwent transcranial Doppler ultrasound (TCD). The statistical correlation between PI or ONSD and ICP 1 week after surgery was analyzed. Furthermore, the areas under the curve (AUCs) of ONSD or PI or a combination of them were calculated to predict intracranial hypertension.

**Results:**

There was a correlation between ONSD and ICP. This correlation still remained at ONSD ≥ 5 mm. Furthermore, there was a strong correlation between PI and ICP. There was a moderate correlation between ICP and PI on days 3, 4, and 5 after surgery (*r* = 0.508, *p* < .001), and a strong correlation on days 6 and 7 after surgery (*r *= 0.645, *p* < .001). Moreover, for predicting intracranial hypertension with PI ≥ 1.2 mm or ONSD ≥ 5 mm or a combination of them, the AUC was 0.729, 0.900, and 0.943, respectively (*p* < .001).

**Conclusions:**

The correlation between ONSD or PI and invasive ICP was different with different levels of ICP in different periods in patients with TBI after surgery. When ONSD ≥ 5 mm and PI ≥ 1.2, it could predict elevated ICP more accurately.

## Graphical abstract

The graphical interface shows scatter plots and linear regression between ICP and ONSD in patients with traumatic brain injury during the first week after surgery. Pearson's correlation indicated highly significant positive strong correlations between them (r = 0.679).

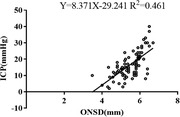

## 1 INTRODUCTION

Invasive intracranial pressure (ICP) monitoring is the gold standard for evaluating ICP in patients with traumatic brain injury (TBI). ICP‐directed therapy, a TBI treatment recommended by the guidelines, can decrease the mortality of severe TBI (Carney et al., 2017; Yuan et al., 2016). The application of ICP is limited because of its complications, including bleeding, iatrogenic infection, bacteria‐free surgical environment, zero drift, and so forth (Tavakoli et al., 2017). As a noninvasive monitoring, transcranial Doppler ultrasound (TCD) monitoring may help to identify cerebral hypoperfusion in patients with TBI who lack invasive ICP monitoring in community hospitals, emergency departments, or intensive care units. Targeted therapy to cerebral blood flow measured by TCD can restore cerebral tissue perfusion within a short time, which is conducive to controlling secondary brain injury (Blanco & Blaivas, 2017; Ract et al., 2007).

The optic nerve sheath is the continuation of the cranial dura mater in the optic canal. The optic nerve sheath diameter (ONSD) in healthy adults was about 2.2–5.0 mm. Increased ICP and enlarged ONSD are independent risk factors of mortality in patients with severe TBI (Zhou et al., 2019). Therefore, ONSD can theoretically reflect the levels of ICP. Currently, there is still a lack of universal ONSD diagnostic criteria for intracranial hypertension. The correlation between ONSD and different ICP levels has also not yet been studied.

Pulsatility index (PI) is an essential index for evaluating the compliance and elasticity of distal cerebral arterioles’ resistance. The calculation formula is PI = (peak systolic velocity − end diastolic velocity) / mean flow velocity. PI is considered to keep pace with invasive ICP measurements when cerebrovascular autoregulation is lost (Kim et al., 2009). Therefore, the increase of ICP may lead to the rise of cerebrovascular resistance, the progressive increase of PI, and the decrease of cerebral blood flow. However, there were different conclusions about the relationship between PI and invasive ICP in different clinical researches, and their correlation at different ICP levels has not yet been studied.

Additionally, there are different physiological theories on the noninvasive assessment of ICP performed by ONSD and PI, which may indicate that the changes of ONSD and PI are not always synchronous with ICP. Moreover, the majority of previous studies in evaluating ICP by ONSD or PI were qualitative rather than quantitative, which was of limited value in guiding treatment. Therefore, we aimed to explore the relationship between ONSD or PI and ICP at different levels or in different periods after surgery, and the ability of ONSD or PI or a combination of them to predict intracranial hypertension in patients with TBI during the first week after surgery.

## 2 PATIENTS AND METHODS

### 2.1 Patients

This retrospective study was approved by the Ethics Committee of the Second Affiliated Hospital of Air Force Medical University (Grant Number: 2018113). A total of 68 patients with TBI, who were treated in the Second Affiliated Hospital of Air Force Medical University between January 2018 and April 2019, were included in this study. Inclusion criteria: (1) patients had closed brain injury; (2) age ≥ 16 years; (3) time from onset to admission ≤4 h; (4) craniectomy with invasive ICP monitoring, high‐quality image of blood flow spectrum, and optic nerve sheath measured by TCD; and (5) the duration of ICP monitoring ≥7 days. Exclusion criteria: patients with a history of craniotomy, cerebral ischemic or hemorrhagic stroke, eyeball or optic nerve injury, endovascular stent implantation for a cephalic and cervical vessel, and open head injury.

### 2.2 Monitoring protocol

All patients received neurocritical care management. The surgery was performed by an associate chief surgeon with 10 years of experience. The surgery procedure was performed as previous described (Carney et al., 2017). The surgeon removed the extradural hematoma, subdural hematoma, and brain contusion during the surgery. The bone flap was removed for the external decompression. ICP, a parenchymal probe (Codman, REF‐826631, Johnson & Johnson Professional Inc., Raynham, MA, USA), was usually placed on the affected side or more severe side of the brain injury to continuously monitor for 7 days after surgery. When ICP ≥ 20 mmHg, the increase of ICP should be considered, and practical strategy should be taken to maintain the ICP < 20 mmHg and cerebral perfusion pressure (CPP) of 60–70 mmHg.

Three qualified sonographers with >5 years of experience conducted TCD at least once a day or whenever the patients had dizziness. The bilateral middle cerebral artery (MCA) was monitored through the temporal ultrasound window using a portable 2‐MHz pulsed TCD device (LOGIQ E9, General Electric Healthcare, Wauwatosa, WI, USA) (D'Andrea et al., 2016). Peak systolic velocity, end diastolic velocity, mean flow velocity, and PI were recorded simultaneously. Abnormal cerebral hemodynamics, including cerebral ischemia, hyperemia, and vasospasm were diagnosed and corrected according to these parameters. The measurement of ONSD was done once a day as follows: The width of the optic nerve sheath was measured 3 mm behind the optic disc with a 7.5–10 MHz ultrasound probe. Each eye was measured twice, and the average value was taken for further analysis. The width of ONSD of 5.00 mm was considered as the critical value of increased ICP.

In general, we evaluated the effectiveness of different monitoring methods at the same time every day. We usually performed TCD first, then evaluated ONSD, and observed the fluctuation of ICP during this period, and the corresponding cerebral blood parameters, ONSD and ICP, were recorded synchronously. For ONSD and TCD, the parameters at the same monitoring site were measured twice at a time, and the average value was taken for further analysis. Continuous monitoring was performed after ICP implantation. If ICP was stable during the period of ONSD and TCD evaluation, the value was recorded. If ICP was changed during this same period, the average was calculated and recorded.

### 2.3 Statistical analysis

SPSS 24.0 (SPSS Inc., Chicago, IL, USA) and MedCalc (MedCalc ver. 19.0.4; MedCalc Inc., Mariakerke, Belgium) were used for statistical analyses. Analysis of agreement between different evaluation methods for ICP was performed using the Bland–Altman statistical method. Counting data were presented as means ± standard deviations (SD), while the measurement data with non‐normal distribution were expressed as medians and interquartile range (P25, P75). The correlation between two variables was also analyzed, and regression analysis was used to describe the estimation of measurable variables to unmeasurable variables. For repeated measures of analysis of variance (ANOVA) of general linear model, Mauchly's test of sphericity was used to test the assumption of sphericity, and the alpha level was set to 0.1. Multiple linear regression described the linear relationship between continuous dependent variables and multiple independent variables. The AUCs of single and multiple factors were used to predict diagnostic sensitivity. The differences between correlation coefficients or AUC values were compared using parametric Z test. All experiments were repeated three times and analyzed by sphericity test. Two‐sided *p*‐values < .05 were considered statistically significant.

## 3 RESULTS

### 3.1 The baseline characteristics

The baseline characteristics of these patients are shown in Table 1. There were 46 males. Their ages were 46.17 ± 16.87 years. The mechanism of injury in patients with ICP included traffic accident injury (75%), falling injury (19.12%), and attack injury (5.88%). Moreover, there were 36.76% unilateral cerebral hernias and 8.82% bilateral cerebral hernias.

**TABLE 1:** Baseline characteristics of the 68 patients with TBI

Characteristic	*N* (%) or median (IQR)
Total number (*n*, %)	68 (100%)
Male sex (*n*, %)	46 (67.64%)
Age (y), mean ± SD	46.17 ± 16.87
GCS on admission, mean ± SD	6.59 ± 2.45
Time from onset to admission (h), median (P25, P75)	7 (5, 18.50)
Time from admission to operation (h), median (P25, P75)	13 (8, 28)
Mechanism of injury (*n*, %)	
Traffic accident injury	51 (75.00%)
Falling injury	13 (19.12%)
Attack injury	4 (5.88%)
Cerebral hernia (*n*, %)	
Unilateral	25 (36.76%)
Bilateral	6 (8.82%)
Cranial CT imaging (*n*, %)	
Cerebral contusion and laceration	35 (51.47%)
Intracerebral hematoma	21 (30.88%)
Subdural hematoma	7 (10.29%)
Epidural hematoma	3 (4.41%)
Subarachnoid hemorrhage	5 (7.35%)
Cerebral infarction	4 (5.88%)

### 3.2 Total number of measurements for ONSD and TCD

In 68 patients, TCD and ONSD monitoring data were recorded 135 times each. After analyzing the TCD image quality, 115 valid data were included in the statistical range. In synchronous monitoring, for ONSD, 86 valid data were finally included for analysis. The details are as follows. (1) For the correlation between ONSD and ICP. For the stratification of ONSD: ONSD < 5 (17 times), ONSD ≥ 5 (69 times). For ICP stratification: ICP < 20 (58 times), ICP ≥ 20 (28 times). (2) For the correlation between PI and ICP. For PI stratification, PI < 1.2 (72 times), PI ≥ 1.2 (43 times). For ICP stratification: ICP < 15 (59 times), 15 ≤ ICP ≤ 20 (32 cases), ICP > 20 (24 times). (3) For the data of ONSD ≥ 5 and PI ≥ 1.2, 30 times were used to analyze the diagnostic intensity of ICP > 20.

### 3.3 The correlation between ONSD and ICP 1 week after surgery

**FIGURE 1:**
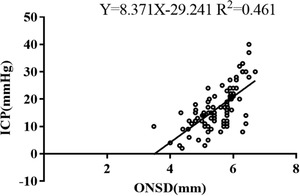
Scatter plots and linear regression between ICP and ONSD

ONSD was strongly correlated with ICP 1 week after surgery (*r* = 0.679, *p* < .001) (Figure 1). Furthermore, there was a strong correlation between ONSD and ICP when ICP was 20 mmHg (*r* = 0.665, *p* < .001), but a weak correlation when ICP was <20 mmHg (*r* = 0.358, *p* = .006). The difference between the two correlation coefficients was statistically significant (Z = 2.066, *p* = .039). Moreover, when ONSD was stratified, there was a strong correlation of ICP with ONSD of ≥5 mm (*r* = 0.644, *p* < .001), but not with ONSD of <5 mm (*p* = .137).

### 3.4 The correlation between PI and ICP 1 week after surgery

**FIGURE 2:**
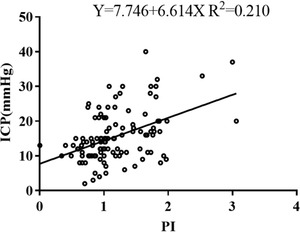
Scatter plots and linear regression between ICP and PI

PI was moderately correlated with ICP 1 week after surgery (*r* = 0.458, *p* < .001) (Figure 2). Moreover, when ICP was stratified, it revealed no correlation of PI with ICP of <15 mmHg (*p* = .366), but a strong correlation with ICP of 15–20 mmHg (*r* = 0.705, *p* < .001) and ICP of ≥20 mmHg (*r* = 0.716, *p *< .001). The difference between the two correlation coefficients was not statistically significant (Z = −0.078, *p* = .938). Furthermore, when PI was stratified, there was a weak correlation between them at PI < 1.2 (*r* = 0.271, *p* = .021) and PI ≥ 1.2 (*r* = 0.350, *p* = .020), respectively. There were no significant differences between these correlation coefficients (Z = −0.440, *p* = .660).

PI was 1.07 ± 0.40 on days 1 and 2 after the operation, 1.16 ± 0.41 on days 3, 4, and 5, and 1.43 ± 0.80 on days 6 and 7. A repeated measure ANOVA test was performed. Mauchlys Test of Sphericity indicated that the sphericity assumption was met (χ^2^ = 2.693, P = 0.260). Tests of within‐subjects effects showed that there was an increasing trend for PI during different periods of time after operation, but no statistically significant difference between them (*F* = 2.830, *p* = .072). There was no correlation between ICP and PI on days 1 and 2 after surgery (*p* = .705), while a moderate relationship between them was found on days 3, 4, and 5 (*r* = 0.508, *p* = .001), and a strong relationship on days 6 and 7 after surgery (*r* = 0.645, *p* < .001. The difference between the two correlation coefficients was not statistically significant (Z = −0.784, *p* = .433).

### 3.5 The ability of ONSD or PI or a combination to predict intracranial hypertension (ICP ≥ 20 mmHg) 1 week after surgery

**FIGURE 3:**
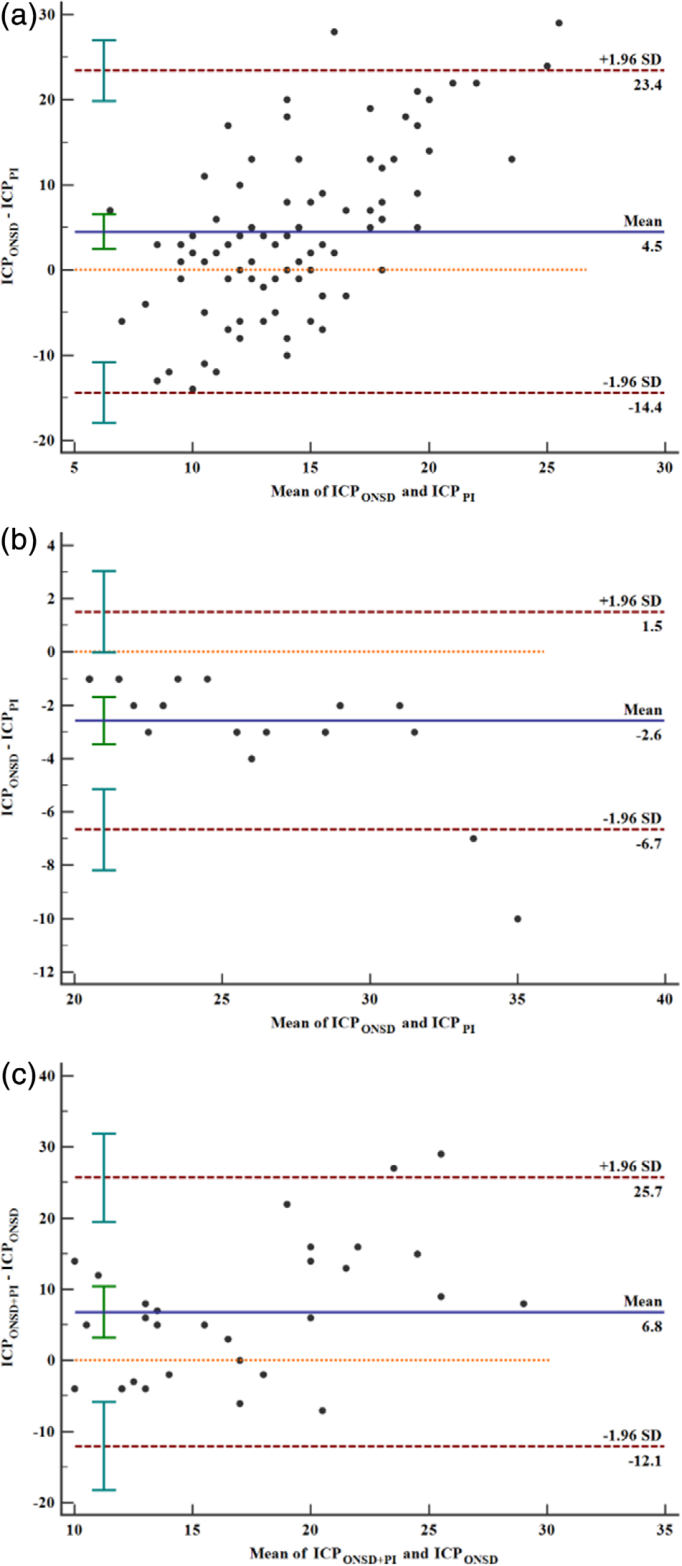
Bland–Altman analysis of agreement. (a) intracranial pressure at ONSD ≥ 5 mm and PI ≥ 1.2, (b) ICP ≥ 20 mmHg at ONSD ≥ 5 mm and PI ≥ 1.2, (c) ICP ≥ 20 mmHg at a combination of ONSD ≥ 5 mm and PI ≥ 1.2 and ONSD ≥ 5 mm alone. Red dotted lines indicate 95% limits of agreement (1.96 SD); the solid blue lines in the middle represent the average value of the difference; the orange dashed lines represent the position where the average value of the difference is 0. There was no specific trend to cause the difference between the two observers. SD, standard deviation

Bland–Altman analysis was performed for the agreement between the different evaluation methods. There was no specific trend to cause the difference between the two observers (Figure 3) (Table 2).

**TABLE 2:** Bland–Altman analysis of agreement

Analysis of agreement	ICP_ONSD_ and ICP_PI_	ICP_ONSD_ and ICP_PI_ at intracranial hypertension	ICP_ONSD + PI_ and ICP_ONSD_ at intracranial hypertension
Mean difference, mmHg (95% CI)	4.48 (2.41–6.55)	−2.58 (−3.46– −1.70)	6.80 (3.20–10.40)
Repeatability coefficient, mmHg (95% CI)	20.75 (18.06–24.39)	6.45 (5.04–8.97)	22.85 (18.26–30.54)
Lower limit, mmHg (95% CI)	−14.43 (−17.98– −10.89)	−6.67 (−8.19 to −5.14)	−12.07 (−18.29 to −5.86)
Upper limit, mmHg (95% CI)	23.39 (19.84–26.94)	1.50 (−0.02–3.03)	25.67 (19.46–31.89)
*p‐*value	<.001	<.001	<.001

**FIGURE 4:**
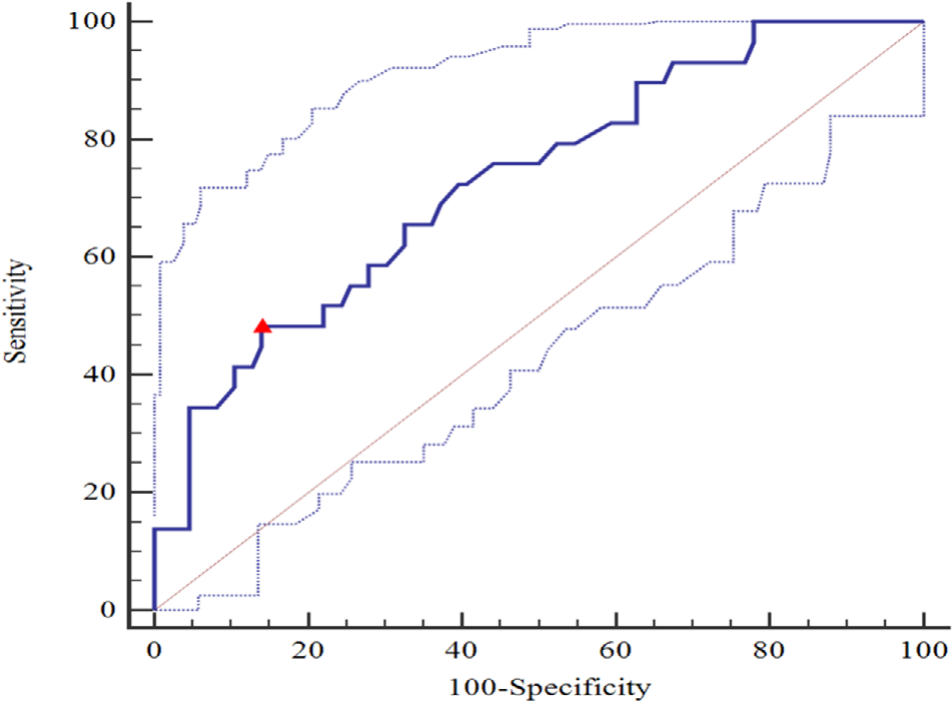
For prediction intracranial hypertension with PI ≥ 1.2, the AUC value was 0.729 (95% CI: 0.637–0.807; *p* < .001). Youden's index 0.343, sensitivity 0.483, specificity 0.860. △, the cut‐off value corresponding to Youden index. AUC, area under the curve; PI, pulsatility index

**FIGURE 5:**
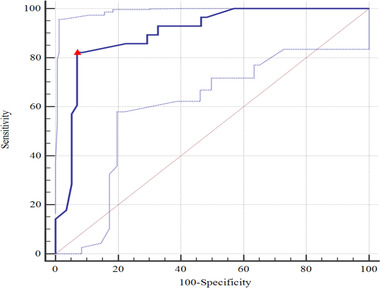
For prediction intracranial hypertension with ONSD ≥ 5 mm, the AUC value was 0.900 (95% CI: 0.816–0.954; *p* < .001). Youden's index 0.752, sensitivity 0.821, specificity 0.931. △, the cut‐off value corresponding to Youden index. AUC, area under the curve; ONSD, optic nerve sheath diameter

**FIGURE 6:**
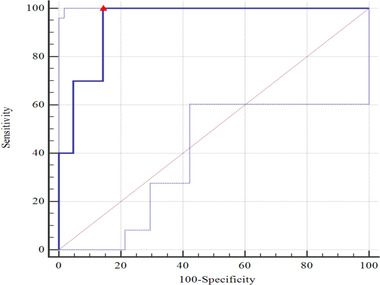
For prediction intracranial hypertension with a combination of ONSD ≥ 5 mm and PI ≥ 1.2, the AUC value was 0.943 (95% CI: 0.796–0.994; *p* < .001). Youden's index 0.857, sensitivity 1.000, specificity 0.857. △, the cut‐off value corresponding to Youden index. AUC, area under the curve; ONSD, optic nerve sheath diameter; PI, pulsatility index

For predicting intracranial hypertension with PI ≥ 1.2 or ONSD ≥ 5 mm alone, the AUC values were 0.729 (95% CI: 0.623–0.834, *p* < .001) (Figure 4) and 0.900 (95% CI: 0.831–0.969, *p* < .001) (Figure 5), respectively, and the difference between the two AUC values was statistically significant (Z = 2.647, *p* = .008). Furthermore, for a combination of ONSD ≥ 5 mm and PI ≥ 1.2 for predicting intracranial hypertension, the AUC value was 0.943 (95% CI: 0.866–1.000, *p* < 0.001) (Figure 6). There was no statistically significant difference between the AUC value of a combination and ONSD ≥ 5 mm alone for predicting intracranial hypertension (Z = −0.819, *p* = .413).

## 4 DISCUSSION

It was believed that the increase of ONSD could quickly and accurately reflect the rise of ICP. Maissan et al. (2015) reported that when ICP increased to more than 20 mmHg during tracheotomy in 18 patients with TBI, ONSD rapidly expanded to more than 5 mm. If the longitudinal measurement of the ONSD width of 5.0 mm was considered to be the diagnostic threshold for intracranial hypertension (Agrawal et al., 2019; Qayyum & Ramlakhan, 2013), it was found that there was a strong correlation between ICP and ONSD 1 week after surgery (*r* = 0.679, *p* < .001). The correlation was stronger at intracranial hypertension than at normal ICP level (*r* = 0.665 vs. *r* = 0.358, *p* = .039). Rajajee et al. (2011) found that ONSD rapidly increased following the increase of ICP. Nevertheless, when ICP returned to normal levels, the ONSD remained to widen. We also found a strong correlation between ICP and ONSD of ≥ 5 mm (*r* = 0.644, *p* < .001), and no correlation when ONSD was < 5 mm (*p* = .137). Therefore, higher ICP corresponded to stronger correlation between ONSD and ICP. When the ICP is decreased, the tension of dura in the cranial cavity is released, but the nerve sheath may still be in the state of expansion. Therefore, when the ICP is reduced or is less than 20 mmHg, ONSD may not allow for the accurate evaluation of the ICP for a weak correlation between them. It suggested that the therapeutic measures based on the decrease of ONSD might prolong the use of osmotic drug or other therapies for ICP management.

So far, there are different conclusions about the relationship between PI and invasive ICP. Bellner et al. (2004) reported that PI was correlated with ICP. When PI was > 2.13 or < 1.2, it was deduced that ICP > 22 mmHg or < 12 mmHg, respectively. Moreover, Prunet et al. (2012) found that TCD‐PI could accurately and effectively predict intracranial hypertension in patients with TBI. The AUC was 0.901, the optimal threshold was 1.35, the sensitivity was 80% and the specificity was 90%. On the contrary, de Riva et al. (2012) argued that TCD‐PI could not accurately predict ICP. It was influenced by CPP, heart rate, arterial pressure difference, cerebrovascular resistance, cerebral artery compliance, and cerebral vascular autoregulation function. The formula was put forward: $PI=\frac{a1}{CPPm}\times \sqrt{{\left(RaCa\right)}^{2}{HR}^{2}{2\pi }^{2}+1}$ (*a*1 was the pressure difference between systolic and diastolic pressure; *CPPm* was arterial pressure; *Ra* was vascular resistance; *Ca* was vascular compliance; and *HR* was heart rate) (Behrens et al., 2010).

In the present study, we found a moderate correlation between ICP and PI 1 week after surgery (*r* = 0.458, *p* < .001). When ICP was stratified, there were no significant differences between these correlation coefficients (*r* = 0.705 vs. *r* = 0.716, *p* = .938). Furthermore, the intensity difference of correlation coefficient between invasive ICP and PI, no matter at PI < 1.2 or PI ≥1.2, was not significant (*r* = 0.271 vs. 0.350, *p* = 0.660). Additionally, the intensity difference of correlation coefficient between ICP and PI at early stage or late stage after surgery was not statistically significant (*r* = 0.508 vs. *r* = 0.645, *p* = 0.433). Therefore, all the findings mentioned above confirmed that PI should be regarded as a dynamic trend of ICP, rather than an absolute value of ICP. PI was not a pressure indicator, which may be affected by the severity of secondary brain injury, cerebrovascular autoregulation, ICP, and other factors (de Riva et al., 2012). Therefore, we should carefully deduce the variation of ICP based on PI in this study. Similarly, it did not mean that the higher ICP led to stronger correlation between invasive ICP and PI.

The regression analysis of ONSD and PI in evaluating intracranial hypertension was carried out in the present study. It showed that AUC value of a combination of ONSD ≥ 5 mm and PI ≥ 1.2 for predicting intracranial hypertension was 0.943. Although there was not a statistically significant difference between the AUC value of a combination and ONSD ≥ 5 mm alone (*p* = .4119), it was a tendency to enhance the ability to predict intracranial hypertension and helped clinicians to change from qualitative to quantitative assessment of ICP (Cardim et al., 2016). This result was consistent with a recent report by Robba et al. (2020) which showed that ONSD was correlated with invasive ICP monitoring better than other noninvasive measurements. Considering the characteristics of those patients and the levels of ICP in this study, we should comprehensively analyze the clinical and imaging examinations before intervention is taken based on PI or ONSD.

There were also several limitations in the present study. First, we were not able to overcome the bias of observational research and small sample size. Second, TCD measurements, including ONSD, were intermittent. However, invasive parenchymal ICP monitoring was continuous, which may have influenced the effectiveness of this study. Third, TCD was performed by different physicians, which may have led to variability in performance and differences in data acquisition. Finally, our results showed different correlations between ONSD and TCD‐PI with ICP, respectively, which did not suggest that these indicators would replace invasive ICP monitoring in patients with TBI.

## 5 CONCLUSIONS

The correlation between ONSD or PI and invasive ICP varies at different levels of ICP and in different periods in patients with TBI after surgery. Additionally, it allows for a more accurate prediction of elevated ICP with a combination of ONSD ≥ 5 mm and PI ≥ 1.2.

## CONFLICT OF INTEREST

The authors declare that they have no potential conflict of interest.

## AUTHOR CONTRIBUTIONS

Tao Chang collected the data and drafted the manuscript. Yanlong Yang and Zhen Qian revised the language and grammar of the manuscript. Zhen Qian and Qingbao Guo provided the clinical data and searched the literature. Lihong Li conceived and designed the experiments. All authors read and agreed to the final manuscript.

### TRANSPARENT PEER REVIEW

The peer review history for this article is available at https://publons.com/publon/10.1002/brb3.2396

## Data Availability

The datasets used and/or analyzed in the present study are available from the corresponding author upon reasonable request.
